# Survey data on Norwegian household energy performance improvement behaviours: Focus on solar PV, flexible energy use, and insulation

**DOI:** 10.1016/j.dib.2025.111930

**Published:** 2025-07-25

**Authors:** Yechennan Peng, Christian A. Klöckner

**Affiliations:** a Department of Psychology, Norwegian University of Science and Technology (NTNU), Norway; b Leibniz-Zentrum für Agrarlandschaftsforschung (ZALF), Eberswalder Str. 84, Müncheberg 15374, Germany

## Abstract

This dataset provides individual-level longitudinal data on energy improvement behaviours, including solar PV adoption, flexible energy use, and home insulation, as well as related psychological variables in Norwegian households. The online survey collected data with participants' consent, covering demographic information, household and housing contextual conditions, psychological responses to the energy crisis, energy-saving behaviours, energy performance improvement behaviours and intentions, and psychological factors influencing decision-making processes for energy-related behaviours. In May 2023, 3,514 participants were recruited from the KANTAR online panel for the first round, and 2,289 of them participated in the second round in November 2023. The paper presents a descriptive summary of most variables, with the results from the two rounds of the survey. This dataset provides valuable insights for researchers exploring residential energy performance improvement behaviours and aids in designing interventions or incentives to reduce residential energy consumption and enhance energy efficiency.

Specifications Table

**Table:** 

Subject	Behaviour science
Specific subject area	Household energy-saving and energy performance improvement behaviours
Type of data	The table was originally created in SPSS (.sav) format and is also available in CSV, STATA (.dta), and R formats
Data format	Raw, filtered, and descriptive data
How data were acquired	Data were acquired through the KANTAR online panel to respondents at least 18 years old and capable of reading Norwegian. The data then were input into SPSS and analysed using Stata.
Description of data collection	It is two-wave longitudinal survey data collected from 3,514 Norwegian households in May and 2,289 of the participants followed the 2nd round survey in November 2023.
Data source location	Data source location: Norway. The survey followed a representative population distribution across Norway’s regions, with specific targets such as Oslo & Viken: 36.3 %; Innlandet: 7.0 %; Agder & Sør-Østlandet: 13.6 %; Vestlandet: 25.3 %; Trøndelag: 8.8 %; Nord-Norge: 9.0 %.
Data accessibility	Repository name: Zenodo Direct URL to all data: 10.5281/zenodo.13355601 Version 1: Original SPSS (.sav) file, and variable description files. 10.5281/zenodo.13355602 Version 2: Converted formats (CSV, STATA [.dta], and R) 10.5281/zenodo.15827534 Version 3: Includes a variable dictionary in both Norwegian and English.* 10.5281/zenodo.15976952

* Note: Norwegian characters (e.g., ø, æ, å) may not display correctly in the webpage preview. To view them properly, download and open the CSV files locally.

## 1 Value of the Data

• Data can be used for structural equation modelling to investigate any of the three energy performance improvement behaviours as well as psychological coping within the decision-making process.
• Data can also be applied in a stage model to explore the drivers and barriers influencing intention transitions within the decision-making process for any of the three energy performance improvement behaviours.
• The data can also facilitate studies examining the spillover effects of energy-saving behaviour.
• The data can also support research on risk-response strategies aimed at enhancing the resilience of the energy system during the energy transition process.
• Based on the identified drivers and barriers of the three energy performance improvement behaviours, this dataset can support the design of tailored subsidy schemes, region-specific programs, and targeted outreach campaigns aimed at underrepresented groups.
• The dataset provides a valuable foundation for developing agent-based models (ABMs) to simulate household behaviour under different policy scenarios, enabling policymakers and researchers to explore the potential impacts of various intervention strategies before implementation.

## 2 Background

**Fig. 1:**
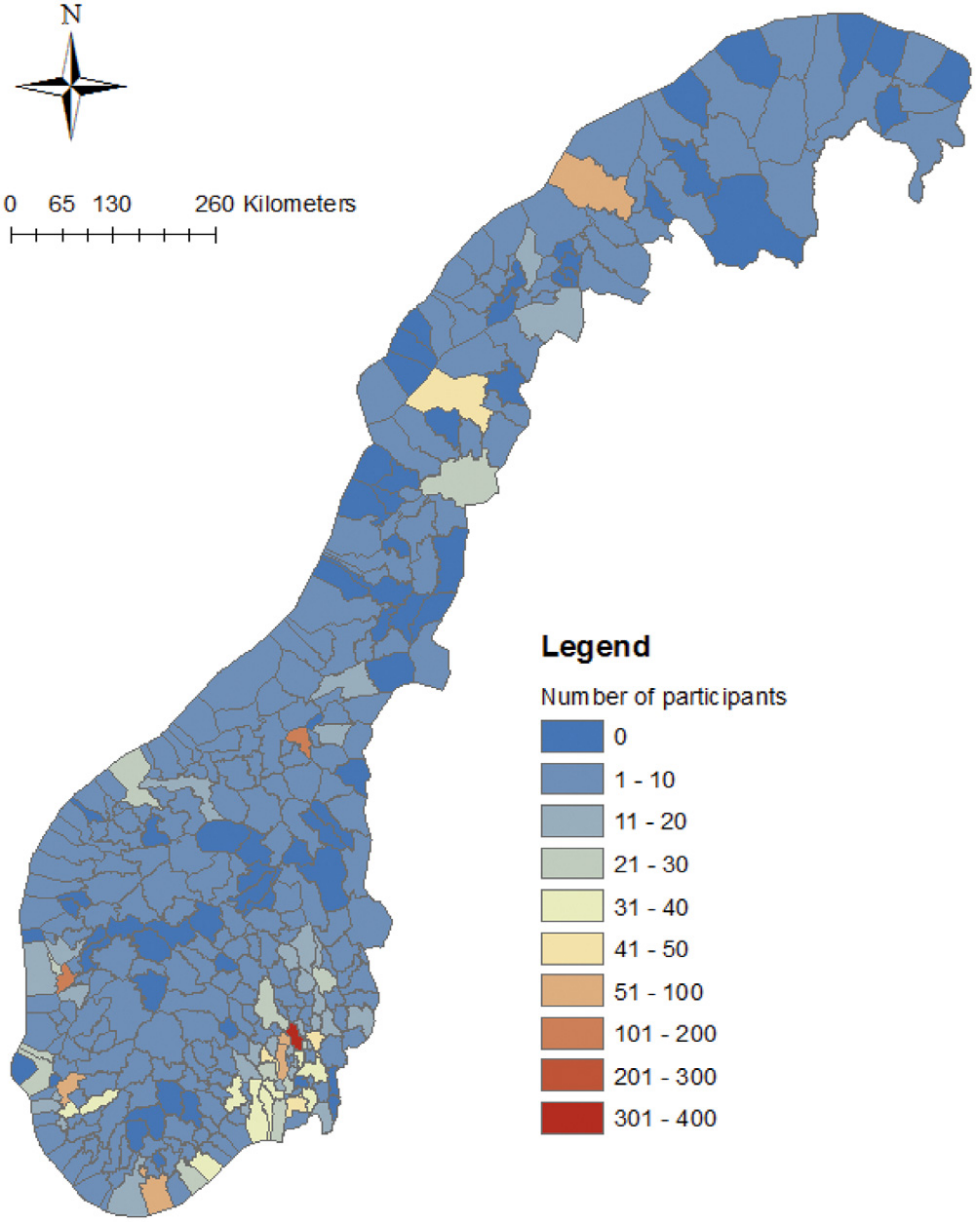
The geographic distribution of participants.

The purpose of this survey is to map the behaviour of Norwegian households in relation to energy retrofitting, solar PV adoption, and flexible energy use practices. The survey builds on earlier work conducted in collaboration with Enova SF—a state-owned enterprise under the Norwegian Ministry of Climate and Environment—focusing on the structural and psychological drivers of home retrofits [[1], [2], [3]]. While the previous survey was conducted in 2014 and 2019, it did not capture the impact of the energy crisis on household behaviour. The present survey was therefore designed to explore longitudinal behavioural changes, with particular attention to the effect of the 2020/21 energy crisis. Fig. 1 shows the geographical distribution of the survey participants. The participants are widely spread across Norway, with the highest concentration (over 300 participants) in the capital city, Oslo. A high number of participants are also found in other major cities, including Bergen and Stavanger along the southern coast, Trondheim in the central region, and Bodø and Tromsø in the north. Only a few remote municipalities, primarily located in sparsely populated areas of the northern and central regions, are not represented in the sample.

### 2.1 Data description

Data in this study informs about Norwegian households' actions related to energy use after energy-crisis with focus on three cases: solar PV (PV case), flexible energy use (Flex case), and retrofitting (Retrofit case). To obtain a representative sample of the Norwegian population, a stratified random sampling method was applied. 3514 Participants were recruited in May and 2289 of the participants followed the 2nd round survey in November 2023. After data cleaning, 2997 valid responses for 1st round survey and 1999 responses for 2nd round survey are retained.

Data include demographic characteristics of the participants, contextual variables of the participants' dwelling, households' conditions, current energy-saving behaviours, and general psychological variables in response to the 20/21 energy-crisis (motivations to save energy, awareness of energy use changes in 2022, and perception of comfort…). Following this, participants were randomly and evenly distributed into one of the three study domains, including solar PV, flexible energy use, and dwelling retrofit. Each domain includes the information of past behaviours, intentions for future actions, general attitudes, personal norms, social norms, descriptive norms, self-efficacy, and other drivers or barriers associated with the performance improvement cases.

Most socio-demographic and contextual variables are measured using continuous values. Some contextual variables are binary, such as whether the respondent is responsible for paying the household electricity bill. All psychological variables, except for comfort perception, are measured on a five-point Likert scale ranging from 1 (strongly disagree) to 5 (strongly agree). The five-point Likert scale was selected for psychological variables as it is commonly used in behavioural research and allows for sufficient variation while being easy for respondents to interpret [4]. Comfort perception is measured on a four-point scale, from 1 (comfortable, not cold) to 4 (very cold). Directly after the energy crisis, a neutral response (e.g. “neither cold nor comfortable”) may have been chosen by many participants to avoid expressing a clear view—particularly on topics with moral or social pressure attached to them. Comfort, in this context, can become socially sensitive due to increasing expectations around energy-saving behaviour. Therefore, for such controversial topics, a response scale without a neutral option was deliberately used to encourage more decisive and meaningful responses [5]. The relevance of this dataset is demonstrated by its use in recent publications [6,7].

Table 1 shows the participants' socio-demographic background, the spatial distribution of the participants, and how many participants were assigned to answer questions related to the three energy performance improvement behaviours.

**Table 1:** Demographic characteristics of participants.

	Norwegian population older than 16 yrs old in 2023 (*n* = 4376,793)		Responders in round1 (*n* = 2,997)		Responders in round2 (*n* = 1,999)	
	**Continuous Variables**					
**Age**						
Mean	48.72		54.53		55.74	
SD	19.02		15.86		15.23	
Skewness	.17		−0.24		−0.29	
Kurtosis	−0.91		2.04		2.12	
	**Categorical variables**					
	n	%	n	%	n	%
**By gender**						
Males	2182,446	49.86	1626	54.25	1148	57.43
Females	2194,347	50.13	1371	45.75	851	42.57
**By Region**						
Oslo	576,792	13.18	400	13.35	262	13.11
Rogaland	379,997	8.68	237	7.91	175	8.75
Møre og Romsdal	213,635	4.88	127	4.24	79	3.95
Nordland	195,364	4.46	146	4.87	90	4.50
Viken	1019,365	23.29	683	22.79	479	23.96
Innlandet	305,877	6.99	197	6.57	126	6.30
Vestfold og Telemark	345,449	7.89	248	8.27	173	8.65
Agder	248,650	5.68	197	6.57	135	6.75
Vestland	511,954	11.70	365	12.18	230	11.51
Trøndelag	382,941	8.75	260	8.68	165	8.25
Troms og Finnmark	196,769	4.50	137	4.57	85	4.25
By education level*						
Primary education	1064,191	23.41	140	4.67	96	4.80
Secondary education	1633,611	35.94	300	10.01	198	9.90
Vocational secondary education			411	13.71	266	13.31
Vocational school	147,053	3.24	316	10.54	222	11.11
Undergraduate education	1153,808	25.38	1020	34.03	664	33.22
Graduate education	546,805	12.03	810	27.03	553	27.66
**By the type of energy performance improvement measures related questions they answered**						
Responders answering question of installing solar PV			937	31.26	641	32.07
Responders answering questions of flexible energy use			971	32.40	647	32.37
Responders answering question of home insulation			1,089	36.34	711	35.57

Note*: The education of Norwegian population includes individuals aged 16 and older [8], while other variables cover those older than 18 only [9].

The data on Zenodo includes the questionnaire in both English and Norwegian, with Norwegian as the original language of the survey, along with participants' answers based on behavioural, socio-demographic, contextual and psychological variables.

## 3 Answers and Measures of Survey Data

### 3.1 Contextual variables

In addition to demographic information, participants were asked about their housing conditions (age, size, and type), household background (income, ownership, type, and years of residence), and electricity payment details (payer, amount paid, and affordability). The electricity payment questions were included only in Round 1, conducted in May 2023, shortly after the energy crisis. Responses, covering both continuous and categorical variables, are shown in Tables 2 and 3.

**Table 2:** Housing and household contextual.

	Responders in round1 (*n* = 2,997)		Responders in round2 (*n* = 1,999)	
	**Continuous Variables**			
**House age**				
Mean	48.80		49.28	
SD	35.33		35.20	
**House size**				
Mean	138.76		140.86	
SD	72.76		72.92	
**Residential year**				
Mean	17.16		17.91	
SD	14.54		14.51	
	**Categorical variables**			
	n	%	n	%
**By household income level**				
<200	26	0.87	16	0.80
200–399	229	7.64	150	7.50
400–599	435	14.51	284	14.21
600–799	450	15.02	300	15.01
800–999	412	13.75	283	14.16
1000–1199	359	11.98	235	11.76
1200–1399	319	10.64	209	10.46
1400–1999	431	14.38	301	15.06
≥2000	336	11.21	221	11.06
**By household type**				
Single family	743	24.79	472	23.61
Two-Adults Family	1377	45.95	954	47.72
One-adult family with a kid	49	1.63	39	1.95
Nuclear family without underage kids	159	5.31	97	4.85
Nuclear Family	220	7.34	144	7.20
Extended family without underage kids	57	1.90	39	1.95
Extended family with underage kids	392	13.08	254	12.71
**By house ownership**				
Owner	2362	78.81	1603	80.19
Shareholder	338	11.28	221	11.06
Short-term rental (<2 yrs)	120	4.00	57	2.85
Long-term rental (>2 yrs)	177	5.91	118	5.90
**By house type**				
Detached House/Villa	1,560	52.05	1,063	53.18
Semi-detached house	195	6.51	136	6.80
Terraced /Chain House	350	11.68	229	11.46
Apartment block	762	25.43	499	24.96
Community residential Block	29	0.97	16	0.80
Basement apartment /Studio	101	3.37	56	2.80

**Table 3:** Housing electricity payment.

	Responders answering question of installing solar PV		Responders answering question of flexible energy use		Responders answering question of home insulation		All responders in round 1	
	Round1 (*n* = 937)		Round1 (*n* = 971)		Round1 (*n* = 1,089)		Round1 (*n* = 2,997)	
**Continuous Variables**								
**Electricity (kWh) used in 2022**								
Mean	16,309.49		16,153.21		16,381.32		16,288.33	
SD	8377.17		8572.95		8232.70		8,379.96	
**Categorical variables**								
	n	%	n	%	n	%	n	%
**Who paid the electricity bill? (who answers yes)**								
Me	772	25.76	803	26.79	939	31.33	2514	83.88
My partner/spouse	338	11.28	352	11.75	369	12.31	1,059	35.34
My children	5	0.17	4	0.13	4	0.13	13	0.43
Other family member	22	0.73	26	0.87	15	0.50	63	2.10
Others outside the family	20	0.67	14	0.47	22	0.73	56	1.87
**Comfort affordability (who answers yes)**								
Did you had money to heat your home to a comfortable temperature?	169	5.64	178	5.94	221	7.37	568	18.95

### 3.2 Psychological variables in response to the 2020/2021 energy-crisis

The psychological variables related to the 2020/2021 energy crisis were mostly measured using a 5-point Likert scale (ranging from “Not at all” to “Very much”), as shown in Table 4. The questions are developed based on the theory of planned behaviour and insights from previous research. [3,10,11]. The exception is the variable “comfort perception,” which was measured using a 4-point Likert scale. Only “awareness of energy use changes” and “motivations,” which are more easily influenced by external factors such as shifts in energy policies, market conditions, or societal awareness, were included in both rounds to capture potential variations over time. Variables related to personal characteristics, past experiences, and personalities, including “acceptance of new measures”, “acceptance of new technology”, and “comfort perception”, were asked only once during Round 1. These variables are considered more stable over time and less likely to be influenced by short-term external changes, making it unnecessary to ask again. For variables (”motivation”, “acceptance of new measures”, and “acceptance of new technology”) assessed through multiple questions, Cronbach's Alpha was tested to evaluate the internal consistency and reliability of the scale, ensuring that the items measured the same underlying construct. In addition, a latent variable was calculated, representing the combined influence of these related items.

**Table 4:** Psychological variables relating to energy-crisis.

	Min.-Max.	Responders in round1 (*n* = 2,997)		Responders in round2 (*n* = 1,999)	
		Mean	SD	Mean	SD
Awareness*					
Are you aware of changes in energy use since winter 2022 (round 1), or since the last time we asked (round 2)?	1–5	4.14	0.81	3.40	0.71
Motivations *					
Motivation to save money by saving energy at home	1–5	4.19	1.00	4.22	0.95
Motivation to protect environment by saving energy at home	1–5	3.17	1.23	3.26	1.22
Motivation to help get through the crisis by saving energy at home	1–5	3.09	1.21	3.12	1.14
Motivation to contribute to security of supply by saving energy at home	1–5	2.90	1.20	3.04	1.13
Motivation to contribute to the energy system for future by saving energy at home	1–5	2.94	1.22	3.04	1.18
Latent variable-Motivation (Cronbach's Alpha)	1–5	3.10 (α=0.83)	1.01	3.18 (α=0.82)	0.97
**Acceptance of new measures**					
I like to try out new ideas.	1–5	3.46	0.92	3.47	0.92
I consider myself creative and original in the way I think and behave.	1–5	3.22	0.97	3.23	0.97
I find it stimulating to be original in the way I think and behave.	1–5	3.06	1.02	3.07	1.01
I often improvise ways to solve a problem when the answer is not obvious.	1–5	3.47	0.92	3.47	0.93
Latent variable- Acceptance of new measures (Cronbach's Alpha)	1–5	3.27 (α=0.79)	0.75	3.28 (α=0.78)	0.75
**Acceptance of new technology**					
I am interested in technology	1–5	3.44	1.10	3.46	1.11
I always want the latest technologies	1–5	2.52	1.10	2.51	1.11
Latent variable- Attitude towards technology (Cronbach's Alpha)	1–5	3.11 (α=0.71)	0.98	3.12 (α=0.70)	0.98
**Comfort perception**					
Have you felt that your home was too cold in the winter of 2022?	1–4	2.13	0.96	2.11	0.97

*Note: These questions were asked in both rounds.

### 3.3 Daily energy-saving measures

The responses to daily energy-saving measures were provided by participants using binary choices (0 for “No” and 1 for “Yes”). Table 5 presents the number and percentage of participants who implemented these energy-saving measures in both rounds.

**Table 5:** Behaviours on daily energy-saving measures.

	Responders in round1 (*n* = 2,997)		Responders in round2 (*n* = 1,999)	
	n	%	n	%
**Energy-saving measures (who answers yes)**				
Reduced indoor temperature	1,404	46.85	559	27.96
Lower indoor temperature when I'm not at home or at night	1,187	39.61	580	29.01
Not heating certain rooms	1,323	44.14	658	32.92
Shorter showers / showered instead of taking a bath	1,330	44.38	511	25.56
Washed clothes at a lower temperature and/or used energy-saving programmes	713	23.79	433	21.66
Avoided using the tumble dryer	781	26.06	381	19.06
Travelled shorter trips with the electric car	358	11.95	60	3.00

### 3.4 Behaviours and intentions

Past energy performance improvement behaviours and future application intentions are assessed by one and one measures. For solar PV, past behaviour specifically refers to all previous private PV installations. In the case of flexible energy use and insulation, past behaviour focuses on actions taken during the three years prior to the Round 1 survey. For Round 2, past behaviours refer to actions undertaken within the six months between May 2023 (Round 1) and November 2023 (Round 2). In this measure, having intention refers to have plan to act within the next three years. The number and percentage of participants who have already performed the behaviours or plan to do so are presented in Tables 6a and 6b.

**Table 6a:** Behaviours and intentions on energy performance improvement measures (round 1).

	Participants in Round 1 (*n* = 2,997) who have taken this action in the past three years		Participants in Round 1 (*n* = 2,997) who plan to take this action in the next three years	
	n	%	n	%
**Solar PV**				
Install private solar PV	21	0.70	58	1.94
Flexible energy use				
Automatically use the dishwasher when electricity is cheapest	26	0.87	131	4.37
Automatically use the washing machine when electricity is cheapest	33	1.10	120	4.00
Automatically use the tumble dryer when electricity is cheapest	15	0.50	87	2.90
Automatically charge your electric car when electricity is cheapest	72	2.40	75	2.50
Automatically delay heating water in the hot water tank when electricity is expensive	31	1.03	303	10.11
Automatically reduce indoor temperature when electricity is expensive, and increase it when electricity is cheap	41	1.37	160	5.34
Automatically heat your cabin (if you have one) based on electricity prices	33	1.10	83	2.77
Automatically use entertainment electronics when electricity is cheap	16	0.53	95	3.17
Automatically spread-out electricity use throughout the day to avoid high consumption peaks	32	1.07	199	6.64
**Retrofitting and insulation**				
Insulate walls	72	2.40	54	1.80
Insulate roof	63	2.10	51	1.70
Insulate floor	34	1.13	24	0.80
Replace windows and doors	103	3.44	73	2.44

**Table 6b:** Behaviors and intentions on energy performance improvement measures (round 2).

	Participants in Round 2 (*n* = 1,999) who have taken this action since we asked		Participants in Round 2 (*n* = 1,999) who plan to take this action in the next three years	
	n	%	n	%
**Solar PV**				
Install private solar PV	11	1.70	37	7.70
Flexible energy use				
Automatically use the dishwasher when electricity is cheapest	15	10.10	80	26.80
Automatically use the washing machine when electricity is cheapest	16	10.70	83	27.80
Automatically use the tumble dryer when electricity is cheapest	10	6.70	49	16.40
Automatically charge your electric car when electricity is cheapest	43	28.90	40	13.40
Automatically delay heating water in the hot water tank when electricity is expensive	20	13.40	199	66.60
Automatically reduce indoor temperature when electricity is expensive, and increase it when electricity is cheap	24	16.10	103	34.40
Automatically heat your cabin (if you have one) based on electricity prices	12	8.10	42	14.00
Automatically use entertainment electronics when electricity is cheap	8	5.40	48	16.10
Automatically spread out electricity use throughout the day to avoid high consumption peaks	19	12.80	127	42.50
**Retrofitting and insulation**				
Insulate walls	14	0.70	42	2.10
Insulate roof	13	0.65	25	1.25
Insulate floor	6	0.30	23	1.15
Replace windows and doors	19	0.95	50	2.50

Intentions are also assessed using an alternative method involving a set of four-stage intention questions that capture participants' self-identified stages in the decision-making process. Bamberg's stage model of self-regulated behavioural change serves as the theoretical framework, based on the premise that rational actors do not change their behaviour in a single step [12]. This four-stage model, adapted to energy-related behaviours, includes: (Stage 1) not being in a decision-making mode, (Stage 2) deciding what to do, (Stage 3) deciding how to do it, and (Stage 4) deciding how to implement the decision [13]. Tables 7a and 7b presents the results in round 1 and round 2, which are derived from participants' self-reported agreement with these intention stages. This agreement measured using a 5-point Likert scale (ranging from “Not true” to “Completely true”). The number and percentage of participants who disagree with the statement (answering “Not true”) are not included in the table, as they do not belong to any stage.

**Table 7a:** Intentions on energy performance improvement measures (round1).

			Slightly true		Partly true		Mostly true		Completely true	
			n	%	n	%	n	%	n	%
Responders answering question of installing solar PV	Round1 (*n* = 937)	Stage 1	34	3.63	8	0.85	1	0.11	14	1.49
		Stage 2	2	0.21	52	5.55	30	3.20	170	18.14
		Stage 3	33	3.52	65	6.94	19	2.03	26	2.77
		Stage 4	47	5.02	36	3.84	8	0.85	17	1.81
Responders answering question of flexible energy use	Round1 (*n* = 971)	Stage 1	6	0.62	6	0.62	3	0.31	6	0.62
		Stage 2	0	0.00	19	1.96	24	2.47	37	3.81
		Stage 3	19	1.96	201	20.70	109	11.23	78	8.03
		Stage 4	47	4.84	258	26.57	67	6.90	47	4.84
Responders answering question of home insulation	Round1 (*n* = 1,089)	Stage 1	68	6.24	50	4.59	9	0.83	31	2.85
		Stage 2	5	0.46	29	2.66	10	0.92	17	1.56
		Stage 3	16	1.47	33	3.03	10	0.92	10	0.92
		Stage 4	79	7.25	80	7.35	22	2.02	35	3.21

**Table 7b:** Intentions on energy performance improvement measures (round2).

			Slightly true		Partly true		Mostly true		Completely true	
			n	%	n	%	n	%	n	%
Responders answering question of installing solar PV	Round 2 (*n* = 641)	Stage 1	34	3.63	8	0.85	1	0.11	14	1.49
		Stage 2	2	0.21	52	5.55	30	3.20	170	18.14
		Stage 3	33	3.52	65	6.94	19	2.03	26	2.77
		Stage 4	47	5.02	36	3.84	8	0.85	17	1.81
Responders answering question of flexible energy use	Round 2 (*n* = 647)	Stage 1	6	0.62	6	0.62	3	0.31	6	0.62
		Stage 2	0	0.00	19	1.96	24	2.47	37	3.81
		Stage 3	19	1.96	201	20.70	109	11.23	78	8.03
		Stage 4	47	4.84	258	26.57	67	6.90	47	4.84
Responders answering question of home insulation	Round 2 (*n* = 711)	Stage 1	68	6.24	50	4.59	9	0.83	31	2.85
		Stage 2	5	0.46	29	2.66	10	0.92	17	1.56
		Stage 3	16	1.47	33	3.03	10	0.92	10	0.92
		Stage 4	79	7.25	80	7.35	22	2.02	35	3.21

### 3.5 Attitude, descriptive norms, social norms, personal norm, and self-efficacy towards energy performance improvement measures

The psychological constructs related to the three energy performance improvement measures, including social norms, personal norms, and self-efficacy, are measured using a 5-point Likert scale ranging from “strongly disagree” to “strongly agree”. Attitudes are also assessed using a 5-point Likert scale, representing a range from negative to positive attitudes. Descriptive norms are measured based on self-reported responses to specific questions. For variables measured through multiple questions, internal consistency and reliability are evaluated using Cronbach's Alpha. However, the responses to the two questions on self-efficacy for flexible energy use and insulation are inconsistent, which hinders the use of a latent variable to represent self-efficacy. Instead, the individual questions can be used for other purposes. Answers are shown in Tables 8a, 8b-8c.

**Table 8a:** Psychological constructs relating to solar PV.

	Min.-Max.	Responders in round1 (*n* = 937)		Responders in round2 (*n* = 641)	
		Mean	SD	Mean	SD
**Attitude**					
Overall, how would you rate the idea of having solar panels on your home, from useless to useful?	1–5	3.51	1.26	3.43	1.22
Overall, how would you rate the idea of having solar panels on your home, from poor to good?	1–5	3.51	1.25	3.45	1.25
Latent variable- Attitude (Cronbach's Alpha)	1–5	3.51 (α=0.93)	1.21	3.44 (α=0.93)	1.20
**Descriptive norms**					
Approximately what percentage of people you know have solar panels on the roof of their home? - %	0–100 %	2.34 %	5.93 %	2.48 %	8.19 %
Approximately, what percentage of your neighbors have solar panels on the roof of their home?- %	0–100 %	1.54 %	5.42 %	1.67 %	5.93 %
Latent variable- Descriptive norms (Cronbach's Alpha)	0–100 %	2.09 % (α=0.71)	5.20 %	2.11 % (α=0.80)	6.74 %
**Self-efficacy**					
I know which person or company to contact to have solar panels installed on my home.	1–5	2.54	1.38	2.58	1.37
I know what I need to do to install solar panels on my home.	1–5	2.62	1.30	2.68	1.33
Latent variable- Self-efficacy (Cronbach's Alpha)	1–5	2.56 (α=0.77)	1.22	2.62 (α=0.78)	1.23
**Personal norm**					
Because of my values/principles, I feel obliged to install solar panels on my home	1–5	2.09	1.12	1.97	1.11
**Social norm**					
People who are important to me think I should install solar panels on my house.	1–5	2.06	1.08	1.95	1.05

**Table 8b:** Psychological constructs relating to flexible energy use.

	Min.-Max.	Responders in round1 (*n* = 971)		Responders in round2 (*n* = 647)	
		Mean	SD	Mean	SD
**Attitude**					
Overall, how would you rate the idea of using energy flexibly in your home, from useless to useful?	1–5	3.62	1.00	3.59	1.04
Overall, how would you rate the idea of using energy flexibly in your home, from poor to good?	1–5	3.62	0.99	3.65	1.02
Latent variable- Attitude (Cronbach's Alpha)	1–5	3.62 (α=0.90)	0.95	3.61 (α=0.89)	0.98
**Descriptive norms**					
Approximately, what percentage of people you know use energy flexibly? - %	0–100 %	35.86 %	26.11 %	35.41 %	26.26 %
Approximately, what percentage of your neighbours use energy flexibly? - %	0–100 %	31.59 %	25.92 %	29.69 %	25.54 %
Latent variable- Descriptive norms (Cronbach's Alpha)	0–100 %	34.38 % (α=0.93)	25.27 %	32.62 % (α=0.91)	24.93 %
**Self-efficacy**					
I know how to get hold of technology that helps me use energy more flexibly.	1–5	2.98	1.14	3.00	1.22
I know what I need to do to use energy more flexibly.	1–5	3.41	1.02	3.46	1.03
Cronbach's Alpha		α=0.61		α=0.62	
**Personal norm**					
Because of my values/principles, I feel obliged to use energy flexibly.	1–5	2.19	1.10	2.17	1.10
**Social norm**					
People who are important to me think I should use energy more flexibly.	1–5	2.35	1.06	2.27	1.05

**Table 8c:** Psychological constructs relating to house insulation.

	Min.-Max.	Responders in round1 (*n* = 1,089)		Responders in round2 (*n* = 711)	
		Mean	SD	Mean	SD
**Attitude**					
Overall, how would you rate the idea of improving insulation your home, from useless to useful?	1–5	3.14	1.25	3.23	1.20
Overall, how would you rate the idea of improving insulation in your home, from poor to good?	1–5	3.31	1.16	3.37	1.14
Latent variable- Attitude (Cronbach's Alpha)		3.21 (α=0.90)	1.15	3.30 (α=0.92)	1.13
**Descriptive norms**					
Approximately, what percentage of people you know have upgraded the insulation in the last 5 years? - %	0–100 %	4.24 %	9.24 %	5.25 %	10.96 %
Approximately what percentage of your neighbours have upgraded the insulation in the last 5 years?- %	0–100 %	4.45 %	14.16 %	4.15 %	12.05 %
Latent variable- Descriptive norms (Cronbach's Alpha)	0–100 %	4.11 % (α=0.70)	8.72 %	4.50 % (α=0.72)	10.52 %
**Self-efficacy**					
I know which person or company I need to contact to have my home professionally insulated.	1–5	2.89	1.34	2.91	1.34
I know what I need to do to insulate my home.	1–5	3.09	1.26	3.21	1.22
Cronbach's Alpha		α=0.63		α=0.60	
**Personal norm**					
Because of my values/principles, I feel obliged to retrofit and improve insulation of my home.	1–5	2.10	1.09	2.09	1.11
**Social norm**					
People who are important to me think I should retrofit and improve insulation of my home.	1–5	1.95	1.08	1.88	1.03

### 3.6 Psychological drivers and barriers for decision-making process

All the questions used to measure the psychological drivers and barriers are based on a 5-point Likert scale ranging from “Strongly Disagree” to “Strongly Agree.” These variables are designed based on previous research aimed at understanding the factors influencing the intention transition process [13]. Tables 9a, 9b-9c shows participants' agreements on these statements.

**Table 9a:** Psychological drivers and barriers for solar PV installations.

	Min.-Max.	Responders in round1 (*n* = 937)		Responders in round2 (*n* = 641)	
		Mean	SD	Mean	SD
Installing solar panels on my home will significantly reduce my energy costs.	1–5	3.23	1.03	3.18	1.07
Installing solar panels increases the market value of my home.	1–5	3.49	1.04	3.44	1.05
Installing solar panels is profitable and will pay back within a reasonable time.	1–5	2.96	1.12	34.08	557.76
Installing solar panels makes my home an overall better place to live.	1–5	2.97	1.04	2.94	1.07
Installing solar panels improves my comfort.	1–5	2.91	0.96	2.77	0.97
I can quickly and easily find information on the practicalities of installing solar panels.	1–5	3.37	1.07	3.29	1.08
I trust the information on solar panel installation I receive from the authorities.	1–5	3.31	1.06	3.32	1.10
There are public support schemes for the installation of solar panels.	1–5	3.61	1.01	3.62	1.02
It is not the right time to install solar panels.	1–5	3.11	1.13	3.19	1.19
Protective regulations prevent me from installing solar panels.	1–5	2.03	1.16	2.05	1.24
I do not have available financial resources (savings or loans) to invest solar panels.	1–5	3.27	1.47	3.29	1.44
The construction companies that could possibly carry out the installation of solar panels are inexperienced and/or lack the necessary knowledge.	1–5	2.68	0.82	2.74	0.84
I have to agree with my neighbours if I want to install solar panels.	1–5	2.65	1.57	2.64	1.62
Installing solar panels creates a lot of disruption and practical disorder in daily life.	1–5	2.33	1.04	2.41	1.03
The installation of solar panels of the house requires a lot of time for the follow-up of the craftsmen.	1–5	3.11	0.93	3.10	0.90
There is a long waiting time for solar panels.	1–5	3.18	0.73	3.14	0.70
I can't get hold of craftsmen who can install solar panels.	1–5	2.87	0.83	2.89	0.80
Installing solar panels spoil the charm of the house.	1–5	2.48	1.23	2.47	1.23
I am considering installing solar cells more in the last year due to higher energy prices.	1–5	2.60	1.32	2.33	1.25
I feel that I am doing a good deed for the energy supply by installing solar panels.	1–5	3.26	1.14	3.26	1.12
I feel I am doing a good deed for the environment by installing solar panels.	1–5	3.32	1.18	3.26	1.15

**Table 9b:** Psychological drivers and barriers for flexible energy use.

	Min.-Max.	Responders in round1 (*n* = 971)		Responders in round2 (*n* = 647)	
		Mean	SD	Mean	SD
Using energy flexibly leads to a significant reduction in my energy costs.	1–5	2.88	1.13	2.82	1.19
Using energy flexibly contributes to more energy security.	1–5	3.11	0.99	3.04	1.06
Using energy flexibly reduces the need to expand the electricity grid.	1–5	3.26	1.12	3.28	1.13
Investing in technology that helps to use energy flexibly increases the market value of my home.	1–5	3.35	1.05	3.33	1.02
I can quickly and easily find information on practical things related to flexible energy use.	1–5	3.20	1.04	3.26	1.05
I trust the information on flexible use of energy I get from the government.	1–5	3.34	1.07	3.41	1.12
I trust the information on flexible use of energy I get from my electricity supplier.	1–5	3.13	1.03	3.17	1.05
I can't decide what to do first when it comes to flexible energy use.	1–5	2.67	1.04	2.56	1.10
It is not yet the right time for more flexible energy use.	1–5	2.87	1.06	2.90	1.13
I do not have available financial resources (savings or loans) to invest in technology that helps with flexible energy use.	1–5	3.07	1.33	3.06	1.35
I have negative experiences from previous attempts to use energy flexibly.	1–5	2.23	1.02	2.17	1.03
There is a long wait for technology that helps with flexible energy use.	1–5	2.89	0.76	2.86	0.81
I can't get hold of craftsmen who can install technology that helps with flexible energy use.	1–5	2.72	0.92	2.71	0.94
Flexible energy use only contributes to the energy industry making more money.	1–5	3.03	1.08	3.05	1.12
Flexible energy use will lead to conflicts in my household.	1–5	2.22	1.15	2.25	1.19
Flexible energy use requires technology that I don't have and don't want to buy.	1–5	3.05	1.06	3.09	1.15
Energy prices are not high where I live.	1–5	1.98	1.18	2.20	1.21
Flexible energy use doesn't fit with my/our daily routines.	1–5	3.27	1.02	3.27	1.09
I feel that I am doing a good deed for energy supply by using energy more flexibly.	1–5	3.26	1.05	3.24	1.11
I feel that I am doing a good deed for the environment by using energy more flexibly.	1–5	3.29	0.95	3.21	1.00

**Table 9c:** Psychological drivers and barriers for retrofitting and insulation.

	Min.-Max.	Responders in round1 (*n* = 1,089)		Responders in round2 (*n* = 711)	
		Mean	SD	Mean	SD
Retrofitting my home will significantly reduce my energy costs.	1–5	3.02	1.12	3.05	1.15
Retrofitting increases the market value of my home.	1–5	3.45	1.13	3.54	1.17
Investing in retrofitting is profitable and will pay back within a reasonable time.	1–5	3.08	1.10	3.11	1.10
Retrofitting insulation has positive health benefits.	1–5	3.25	1.00	3.35	1.05
The standard of my current home insulation is a waste of energy.	1–5	2.56	1.24	2.56	1.22
Retrofitting makes my home an overall better place to live.	1–5	3.34	1.15	3.43	1.17
Retrofitting insulation improves comfort.	1–5	3.59	1.05	3.70	1.05
I can quickly and easily find information on the practicalities of retrofitting my home.	1–5	3.35	1.08	3.45	1.09
I trust the information I receive from the authorities about retrofitting.	1–5	3.35	1.03	3.39	1.00
There are public support schemes for retrofitting homes.	1–5	3.18	1.07	3.21	1.04
I can't decide what to do first when it comes to retrofitting.	1–5	2.32	1.12	2.31	1.12
It is not yet the right time to retrofit my house.	1–5	3.70	1.19	3.76	1.17
Building protection regulations prevent me from carrying out retrofitting.	1–5	1.71	1.01	1.60	0.99
I do not have available financial resources (savings or loans) to invest in retrofitting.	1–5	3.08	1.40	3.12	1.40
The construction companies that could possibly carry out the retrofitting are inexperienced and/or lack the necessary knowledge.	1–5	2.35	0.97	2.30	1.01
I have to agree with my neighbours if I want to retrofit my house.	1–5	2.17	1.48	2.19	1.54
A retrofitting project creates a lot of disruption and practical disorder in daily life.	1–5	3.09	1.18	3.11	1.21
Retrofitting your home requires a lot of time to follow up on the workmen.	1–5	3.18	1.11	3.19	1.08
I have negative experiences from previous retrofitting projects.	1–5	1.84	1.01	1.79	1.01
There are long waiting times for building materials.	1–5	2.65	0.91	2.58	0.92
I can't get hold of craftsmen	1–5	2.10	1.04	2.08	1.04
Upgrading will ruin the charm of the house	1–5	1.85	1.04	1.79	1.01
I feel that I am doing a good deed for the energy supply by implementing measures to improve the energy standard of my home.	1–5	3.05	1.11	3.08	1.08
I feel that I am doing a good deed for the environment by taking measures to improve the energy performance of my home.	1–5	3.13	1.12	3.11	1.14

## 4 Experimental Design, Materials and Methods

The survey is a follow-up of previous survey—focusing on the structural and psychological drivers of home retrofits [[1], [2], [3]]. The variables used were derived from an extensive literature review [1] and are informed by established psychological theories such as the Theory of Planned Behaviour [2] the Norm-Activation-Theory [3] or the Comprehensive Action Determination model [4]. Survey questions of a similar structure were also adapted to examine household investment behaviour regarding private solar PV and the adoption of flexible energy use practices. All participants were recruited from Kantar's online survey panels, a platform consisting of pre-profiled individuals who have consented to take part in surveys. The panel is used to collect data on a wide range of topics through ad hoc survey studies.

Stratification was applied based on key demographic and geographic criteria, including region, gender, and age, with quotas aligned to national population statistics provided by Statistics Norway (SSB, 2022: https://www.ssb.no/statbank/table/07459/). Forst, the target sample consisted of individuals aged 18 and above who were able to read Norwegian. The intended age distribution was approximately 45 % aged 18–44, 35 % aged 45–66, and 20 % aged 67 or older. Second, the sample also aimed for a representative gender distribution, targeting approximately 50.2 % male and 49.8 % female respondents. Third, the sampling ensured regional representation across six areas (e.g., (Oslo & Viken: 36.3 %; Innlandet: 7.0 %; Agder & Sør-Østlandet: 13.6 %; Vestlandet: 25.3 %; Trøndelag: 8.8 %; Nord-Norge: 9.0 %), and aimed for a total of at least 3000 completed responses across three cases of the study. Stratification was applied based on Acceptable deviations were predefined (±2 % for region, ±3 % for gender, and ±7 % for age due to recruitment challenges in the oldest age group).

The survey data were carefully examined for completeness and internal consistency. Respondents with substantial missing data were excluded from the final dataset. For individual variables with minor missingness, values were either left as missing or filled in when they could be reliably inferred from other responses. For example, if a respondent did not specify whether they were an owner or renter but indicated they were renting their current dwelling and planned to move out within the next two years, it was reasonable to infer that they were a renter. In such cases, missing values were completed accordingly. Additionally, cases containing implausible or internally inconsistent responses were excluded. For instance, if a participant reported a dwelling size of approximately 200 m² but also answered “yes” to the question of whether their home was larger than 500 m², the inconsistency rendered the data unreliable, and the case was removed from the dataset.

## Limitations

Some limitations should be considered when using the dataset. First, the dataset overrepresents older and more educated individuals, potentially limiting the generalizability of the findings to younger or less educated groups. The dataset tends to overrepresent more educated and older individuals, which may limit the generalizability of findings to younger or less-educated populations. Second, while the dataset includes many participants from remote municipalities, these are spread across different areas, resulting in a lower number of responses per individual remote municipality. In contrast, centralized municipalities (e.g., Oslo) are more densely represented. This imbalance may affect the ability to accurately capture the local context or behavioural patterns within specific remote municipalities. Third, the data are based on self-reported responses, which are subject to recall bias and social desirability effects. Finally, although the dataset includes two survey rounds from 2023, the time span may not fully capture long-term behavioural trends or responses to evolving policy and market conditions. For studies aiming to investigate the long-term development of energy retrofitting practices, this dataset should ideally be used in conjunction with earlier nationally representative surveys on private housing energy retrofitting in Norway—which was conducted January and March 2014, and between March and April 2019 [[1], [2], [3]]. These earlier datasets are openly available via Mendeley Data (DOI: 10.17632/vmyn94prrr.1), provided by Lars Even Egner and Christian A. Klöckner [14].

## List of Abbreviations and Terms

**Table:** 

Abbreviations / Terms	Definitions
PV	Photovoltaic
KANTAR online panel	The platform consists of pre-profiled individuals who have consented to take part in surveys
Enova SF	A state-owned enterprise under the Norwegian Ministry of Climate and Environment
Awareness	Awareness of changes in energy use
Motivations	Motivations for saving energy at home
Acceptance of new measures	Individual's openness to explore original and improvised solutions.
Acceptance of new technology	Individual's interest in and enthusiasm for adopting the latest technological innovations.
Comfort perception	Individual's subjective experience of indoor thermal comfort
Attitude	Individual's overall evaluation of measures on their home,
Descriptive norms	Individual's perception of how common measures adoption is among people they know
Self-efficacy	Individual's confidence in their ability to take the necessary steps of measures
Personal norm	Individual's sense of moral obligation of performing in the measures
Social norm	Individual's perceived social pressure from important others

## Ethics Statement

The project received an approval from the Norwegian university of technology and science Institutional Review Board. It also followed all of the APA ethical guidelines. The data processor collects all data for the study, recruits participants and contacts them again for round 2 of data collection. Before data is sent to the data controller, email addresses are replaced with an anonymous code that allows wave 1 and wave 2 of data collection to be linked without sending personal data to the data controller. Participation in the questionnaire was voluntary, and no personal information, such as names or identities, was collected to ensure respondents' privacy. Hence, participant responses were completely anonymous. The reference number for this project is 830,673.

## Declaration of Generative AI and AI-Assisted Technologies in the Writing Process

During the preparation of this work, the authors used ChatGPT 4.0 to improve language and readability. After using this service, the authors reviewed and edited the content as needed and take full responsibility for the content of the publication.

## CRediT authorship contribution statement

**Yechennan Peng:** Conceptualization, Methodology, Formal analysis, Investigation, Validation, Writing – original draft. **Christian A. Klöckner:** Conceptualization, Resources, Validation, Writing – review & editing.

## Data Availability

ZenodoSurvey data on norwegian household energy use, with focus on solar PV, flexible energy use, and retrofitting in 2023 (Original data).
